# Assessing the effectiveness and cost-effectiveness of a solution-focused resource-orientated approach (DIALOG+) to improving the quality of life for people with psychosis in India and Pakistan—a cluster RCT

**DOI:** 10.1186/s13063-022-07032-y

**Published:** 2023-01-26

**Authors:** Victoria Jane Bird, Sana Zehra Sajun, Renata Peppl, Sara Evans-Lacko, Stefan Priebe, Swaran Singh, Lakshmi Venkatraman, Padmavati Ramachandran, Aneeta Pasha, Ashar Malik, Onaiza Qureshi

**Affiliations:** 1 Queen Mary University of London, London, UK; 2 London School of Economics and Political Science, Care Policy and Evaluation Centre, London, UK; 3 Warwick University, Coventry, UK; 4 Schizophrenia Research Foundation, Chennai, India; 5 Interactive Research and Development, Karachi, Pakistan; 6 Agha Khan University, Karachi, Pakistan

**Keywords:** Global mental health, Psychosocial interventions, Resource-oriented approach, LMICs, Solution-focused, Quality of life

## Abstract

**Background:**

Severe mental illness (SMI) presents a major challenge worldwide, affecting approximately 5–8% of the world’s population. It causes significant distress to affected people, families and wider communities, generating high costs through loss of productivity and ongoing healthcare use. Over 75% of patients with psychosis receive inadequate care and experience a negative financial impact and reduced quality of life (QoL). It is therefore a priority to reduce the treatment gap by providing low-cost, effective interventions for people with psychosis.

Our research project, PIECEs, is designed to explore, adapt and test a low-cost, approach (DIALOG+) that makes use of existing resources to improve community-based care for patients with psychosis. The research will be conducted in two urban sites: Karachi, Pakistan and Chennai, India. DIALOG+ is a novel, technology-assisted and resource-oriented intervention, based on QoL research, concepts of patient-centred communication, IT developments and solution-focused therapy. However, the approach has not been rigorously tested within India and Pakistan. Our randomised controlled trial (RCT) aims to test the effectiveness and cost-effectiveness of DIALOG+ in improving the QoL and clinical outcomes for individuals with long-term psychosis being treated in the community in India and Pakistan.

**Methods:**

To assess the acceptability, feasibility, and cost effectiveness of DIALOG+, we will conduct a cluster RCT with 210 patients and 14 clinicians in each country. The intervention will be used during a routine interaction between a clinician and a patient. It consists of a patient-centred assessment (the DIALOG scale) whereby the clinician invites the patient to rate their satisfaction with different life domains and treatment aspects, which forms the active control group. The intervention group will follow this up with a four-step solution-focused approach to identify the patient’s resources and develop solutions to deal with the patient's concerns (DIALOG+).

**Discussion:**

If shown to be effective DIALOG+ has the potential to improve community-based care and the QoL for millions of people within India and Pakistan who experience psychosis.

**Trial registration:**

The trial was registered prospectively on the ISRCTN Registry: ISRCTN13022816 on 9 February 2022.

## Administrative information

Note: The numbers in curly brackets in this protocol refer to SPIRIT checklist item numbers. The order of the items has been modified to group similar items (see http://www.equator-network.org/reporting-guidelines/spirit-2013-statement-defining-standard-protocol-items-for-clinical-trials/).

**Table:** 

Title {1}	Assessing the effectiveness and cost-effectiveness of a solution-focused resource-orientated approach (DIALOG+) to improving the quality of life for people with psychosis in India and Pakistan - a cluster RCT.
Trial registration {2a and 2b}.	ISRCTN Registry Number: ISRCTN13022816
Protocol version {3}	Protocol version: V1.0, 15^th^ Jan 2022
Funding {4}	This research was funded by National Institute of Health Research (NIHR-funded) Research and Innovation for Global Health Transformation (RIGHT) programme (NIHR200824) using UK aid from the UK Government to support global health research. The views expressed in this publication are those of the author(s) and not necessarily those of the NIHR or the UK Department of Health and Social Care.
Author details {5a}	Victoria Jane Bird, Sana Zehra Sajun, Renata Peppl, Stefan Priebe – *Queen Mary University of London, UK* *Sara Evans-Lacko – London School of Economics and Political Science, Care Policy and Evaluation Centre, UK* *Swaran Singh – Warwick University, UK* *Lakshmi Venkatraman**, Padmavati Ramachandran – Schizophrenia Research Foundation, India* Aneeta Pasha, Onaiza Qureshi – *Interactive Research and Development, Pakistan* Ashar Malik *– Agha Khan University, Pakistan*
Name and contact information for the trial sponsor {5b}	Joint Research and Management Office (JRMO) Queen Mary University of London Mile End Road London, E1 4NS
Role of sponsor {5c}	The sponsor and funder play no role in study design; collection, management, analysis, and interpretation of data; writing of the report; and the decision to submit the report for publication.

## Introduction

### Background and rationale {6a}

Severe mental illness (SMI) presents a major challenge worldwide, affecting approximately 5–8% of the world’s population [1, 2]. SMI causes significant distress to affected people, families and wider communities, generating high costs through, for example, loss of productivity and ongoing health and social care use [3]. Within low- and middle-income countries (LMICs), there are neither sufficient financial resources nor qualified staff to provide extensive specialised services to people with SMI. As a result, an estimated 69–89% of people with SMI within LMICs experience a treatment gap [4, 5]. This treatment gap is most pronounced for psychosis, particularly long-term psychosis, where 75% of all individuals do not receive adequate care, despite the high financial impact and reduced quality of life (QoL) [3, 4]. It is therefore an urgent priority to reduce this treatment gap by providing low-cost, effective interventions for people with long-term psychosis [6].

The prevalence of mental health conditions in Pakistan is reportedly up to 34% [7]. Inadequate attention to mental health in the public sector has resulted in an estimated ratio of 2–3 psychiatrists per million people [8, 9]. A lack of mental health services and widespread poverty mean mental health issues often remain undiagnosed, misdiagnosed or untreated. In India, the estimated lifetime prevalence of mental disorders is 13.9% [10], with inadequate infrastructure, financial and human resources resulting in a ratio of 3 psychiatrists per million people. Care is reported to be inadequate for 70–75% for patients with psychosis in India [11].

Resource scarcity within healthcare systems in LMICs has led to calls for more stringent priority setting and allocation of available resources to ensure the most effective use. In high-income countries (HICs), evidence on the cost-effectiveness of innovative approaches to manage mental health conditions is routinely generated to inform policy makers. Studies in HICs confirm that community management of psychosis represents the best value for money and can result in future resource savings for the healthcare system [12–14].

A large proportion of people with psychosis in India and Pakistan live with their families as there are limited alternatives and welfare support [15]. Any care for people with psychosis is predominantly managed by limited conventional inpatient facilities, where patients can stay for long periods removed from their social context and without effective psychosocial interventions. A lack of qualified staff can limit the ability to provide services used in HICs such as multi-disciplinary teams or specialised psychological treatments (e.g. cognitive behaviour therapy). There is therefore a need for effective, appropriate and low-cost forms of care that utilise and strengthen existing personal and social resources available to individuals, families and communities.

This RCT sits in a wider programme of research, the PIECEs project, which aims to improve community services for people with long-term psychosis by adapting, testing and implementing a low-cost, approach called DIALOG+. The programme and RCT is being conducted in two urban cities: Karachi, Pakistan and Chennai, India. Approaches to community care shown to be effective here will have a high chance of success in other sites across the Indian subcontinent and, if successful, there is potential to improve community-based care and the QoL for millions of people within India and Pakistan who experience psychosis.

Previous research developed DIALOG+ which is an evidence-based approach to improve the quality of care for people with long-term psychosis, by utilising the existing resources available to patients within their families, community and healthcare services. DIALOG+ is a novel technology-assisted and resource-oriented intervention that is based on QoL research, concepts of patient-centred communication, IT developments and solution-focused therapy [16, 17]. The approach makes use of existing meetings between patients and clinicians, making them more effective. However, the approach has not been rigorously tested within India and Pakistan. This RCT aims to test the effectiveness and cost-effectiveness of DIALOG+ in improving the QoL and clinical outcomes for individuals with long-term psychosis being treated in the community in India and Pakistan.

### Objectives {7}

The cluster RCT has three main aims:

1. To test the effectiveness of DIALOG+ as compared to an active control in improving the quality of life, clinical and social outcomes for individuals with long-term psychosis receiving community-based care in India and Pakistan
2. To assess the cost-effectiveness of DIALOG+ from the healthcare perspective
3. To understand the experience and acceptability of DIALOG+ within routine services in India and Pakistan

### Trial design {8}

To test DIALOG+ within routine healthcare settings in India and Pakistan we are conducting a cluster RCT. The design of the cluster RCT was finalised after incorporating the results of a pilot study which informed local adaptation of the intervention.

The unit of analysis for the cluster RCT will be the mental health professionals, who will be recruited from outpatient clinics at three included clinical sites (two in Pakistan and one in India). The caseloads of the mental health professionals will be screened by researchers to identify potentially eligible patients. The clinician along with their enrolled patients will form individual clusters and will be allocated on a 1:1 basis to either the control (DIALOG scale) or the intervention arm (DIALOG+).

Assessors will be blinded to participant allocation for all data collection.

## Methods: participants, interventions and outcomes

### Study setting {9}

The research will be conducted in two urban settings: one outpatient clinic in Chennai, India (Schizophrenia Research Foundation - SCARF), and two facility centres in Karachi, Pakistan—a public-sector tertiary hospital (Jinnah Postgraduate Medical Centre—JPMC) and an outpatient clinic at a private mental health hospital (Karwan-e-Hayat- KeH).

### Eligibility criteria {10}

Inclusion criteria:

Individuals with psychosis

- Aged 18–65 years old
- Diagnosis of psychosis defined as an ICD-10 diagnosis of Schizophrenia, schizotypal, delusional and other non-mood psychotic disorders (F20-29) and/or bipolar disorder with psychotic features (F31.2, F31.5, F31.64)
- Currently not receiving inpatient treatment
- Duration of illness greater than 2 years
- Score < 5 on Manchester Short Assessment of Quality of Life (MANSA)
- Capacity to provide informed consent based on an adapted ‘University of California, San Diego Brief Assessment of Capacity to Consent (UBACC)’ Scale score of 12 or above
- Ability to speak and understand the local language; Urdu (Pakistan), Tamil (India) or English

Clinicians

- Aged 18 years or over
- Has regular clinical contact with individuals with psychosis
- Experience of working with individuals with psychosis
- No plans to leave the current post within the next 6 months
- Ability to speak and understand the local language; Urdu (Pakistan), Tamil (India) or English

Family members/carers

- Primary caregiver of a person with psychosis enrolled in the DIALOG+ RCT (primary caregiver defined as the main person responsible for helping with activities of daily living, supporting, and advocating on behalf of the patient)
- Has been the primary caregiver of a person with psychosis for more than 6 months
- Aged 18–75 years old
- Ability to speak and understand Urdu (Pakistan), Tamil (India) or English

Exclusion criteria:

Individuals with psychosis

- Dementia and/or significant cognitive impairment cognitive impairment and/or severe learning disability
- Organic psychosis or drug-induced psychosis if given as the primary diagnosis
- Unable to provide informed consent on the UBACC scale (score < 12)

Clinicians, family members/carers

- Does not have regular contact with individual(s) with chronic psychosis
- Unable to speak either English, Urdu or Tamil

#### Who will take informed consent? {26a}

Potentially eligible patients will be approached by their clinicians to introduce the study to them. Individuals who respond to the study information with interest will be contacted and invited to attend a face-to-face meeting with a researcher who will provide them with further information. Researchers will go through consent forms (in their preferred language) with interested individuals and take time to answer any questions or concerns that are raised. All participants are asked to provide informed consent by signing and dating an informed consent form prior to any data collection commencing. The University of California, San Diego Brief Assessment of Capacity to Consent (UBACC) instrument will be used to rate the ability of individuals to provide informed consent. This form was adapted to our consent form and a score of 12 or above qualifies the patient to take part in the RCT. Once the ability to provide informed consent has been ascertained, participants will be provided with two copies of the written consent form, which will be signed by the participant and a member of the research team in order to proceed with study participation. The participant will keep one copy of the informed consent form and the research team will keep the other, storing it in a locked filing cabinet. Where participants are not literate, an additional witness will also be required to sign the consent form.

All researchers have received training based on Good Clinical Practice by members of the UK-based research team, senior members of the local research team and/or through online courses. If there are any doubts regarding the individual’s capacity to consent to take part in research, this will be resolved before proceeding with study participation. If any doubts about their capacity emerge during the recruitment process, or capacity to consent appears to change during their participation in the study, their capacity to consent will be re-evaluated before continuing with study participation.

#### Additional consent provisions for collection and use of participant data and biological specimens {26b}

Additional consent provisions are not applicable as no biological samples will be collected.

### Interventions

#### Explanation for the choice of comparators {6b}

In order to control for the novelty of a tablet computer within the consultation, and to control for the repeated measurement of quality of life, an active comparator (DIALOG scale) will be used within the trial. The DIALOG scale comprises 11 items made up of eight life domains (mental health, physical health, job situation, accommodation, leisure activities, friendships, relationship with family/partner, personal safety) and three treatment aspects (medication, practical help, meetings with professionals). Each item is rated on a scale from 1 (‘totally dissatisfied’) to 7 (‘totally satisfied’) and followed by a question on whether the patient wants additional help with that domain. Unlike the DIALOG+ intervention, clinicians will be instructed to ask participants to complete the scale at the end of the session, without further discussion and use of the four-stepped solution focused approach.

#### Intervention description {11a}

The intervention being tested is an evidence-based app mediated intervention called DIALOG+. The intervention and its training methodology has been adapted during a pilot phase and is now being tested within a cluster RCT.

DIALOG+ is used during a routine interaction between a clinician and a patient. It aims to better utilise the resources of the healthcare system by making these meetings more effective and by mobilising other existing resources available to the patient. It consists of a patient-centred assessment (the DIALOG scale) whereby the clinician invites the patient to rate their satisfaction with different life domains and treatment aspects. This is followed by a four-step solution-focused approach to identify the patient’s resources and develop solutions to deal with the patient's concerns. The intervention is available as an app and makes use of a tablet computer (e.g. iPad or Android device) within routine clinical meetings. Clinicians will receive training from the researchers in how to use the intervention, including how to support patients who are unfamiliar with a tablet computer.

Each session begins with the patient using the tablet to rate their satisfaction with eight life domains (mental health, physical health, job situation, accommodation, leisure activities, friendships, relationship with family/partner, personal safety) and three treatment aspects (medication, practical help, meetings with professionals). The tablet allows patients to be more actively involved in the meeting, with the tablet easily passed between the clinician and patient. Each satisfaction item is rated on a scale from 1 (‘totally dissatisfied’) to 7 (‘totally satisfied’) and followed by a question on whether the patient wants additional help with that domain.

The ratings are then summarised on screen, allowing for comparisons with ratings from previous meetings. Clinicians are instructed to offer positive feedback on any improving or high-scoring domains. The ratings are followed by a four-step solution-focused approach to identify the patient’s existing resources that can be used to address the concerns raised. The four steps are as follows: Understanding (Why is the patient dissatisfied? What went nevertheless well?); Looking Forward (What is the best case scenario? What is the smallest step forward?); Exploring Options (What can the patient, the clinician or others do?); and finally Agreeing on Actions (e.g. homework and referrals).

The intervention (both the DIALOG scale and DIALOG+) have already been translated into 16 different languages and for this particular trial, the app has been translated and adapted to use Urdu and Tamil script in Pakistan and India respectively.

Both the DIALOG scale in the control group DIALOG+ in the intervention group will be used once a month for 6 months which forms the active intervention period. Patients and clinicians will be actively followed up during this timeframe to ensure the sessions take place. After this period, there will be a 6-month flexible period where patients and clinicians can decide to use the intervention as per their discretion. This period will allow us to assess sustainability without researcher involvement and routine implementation of the DIALOG+ intervention.

#### Criteria for discontinuing or modifying allocated interventions {11b}

Although the intervention to be tested, DIALOG+, does not pose any risk to participants as evidenced by previous research, participants may experience anxiety in trying new interventions. Individuals will continue to receive their routine care, including any medication and psychosocial interventions that may be available. Furthermore, the intervention (DIALOG+) can be stopped at any point.

#### Strategies to improve adherence to interventions {11c}

Research coordinators will be assigned to monitor routine DIALOG+ appointments for all participants for the first 6 months of the intervention period. Researchers will actively assign appointments for the intervention/control sessions with participants and conduct reminder calls before a patient’s appointment to ensure maximum attendance.

The research teams will record attendance for all meetings in a ‘Meeting Record Log’ and data from the DIALOG+ app will be extracted on a monthly basis to monitor the duration and other details of the meeting.

To monitor further intervention fidelity, a random selection of sessions will be audio-recorded, and the DIALOG+ fidelity scale applied to measure adherence to the intervention and manual. Participants (both clinicians and patients) will be asked at the point of informed consent for additional consent to record sessions. It will be made clear to participants that this is optional, and consent will be confirmed prior to any sessions being recorded.

#### Relevant concomitant care permitted or prohibited during the trial {11d}

Individuals within both the control and intervention arms will continue to receive their usual care during the trial period. This includes access to routine services such as psychosocial interventions (where provided) and pharmacological interventions. We will, however, exclude individuals who are currently taking part in other RCTs of psychosocial interventions being conducted at the sites to reduce the potential for contamination.

#### Provisions for post-trial care {30}

It is highly unlikely that there will be any kind of harm suffered as a result of trial participation. In the very unlikely event that a participant discloses information regarding immediate risk to the self or others, their participation in that session will be immediately terminated and one of the researchers will inform the relevant safeguarding authorities (e.g. clinic). All researchers will be trained in safe-guarding procedures and will be provided with on-going supervision in policies and procedures for, and in working with, individuals who disclose risks of harm. All research activities will be completed by trained and qualified researchers with experience of working with people with mental health difficulties.

### Outcomes {12}

Quantitative outcome measures will be collected at 3 different timepoints: baseline, 6 months post randomisation and 12 months post randomisation. The primary outcome will be quality of life assessed via the Manchester Short Assessment of Quality of Life (MANSA) [18] score at 6 months post randomisation. Mean scores 6-month post randomisation will be calculated controlling for baseline scores and then compared between the two groups to assess the impact of the intervention.

Secondary clinical outcome measures include the psychiatric symptom severity score via Brief Psychiatric Rating Scale (BPRS) [19], negative symptom severity score via the Scale for Assessment of Negative Symptoms (SANS) [20] and disability measure via World Health Organization Disability Assessment Schedule (WHODAS) by proxy [21].

Secondary social outcome measures include the objective social situation score via Social Situation Index (SIX) [22] and therapeutic alliance for patients and clinicians via the Helping Alliance Survey (HAS) [23] score. The Burden Assessment Schedule [24] (in Pakistan) and The Burden Assessment Scale [25] (in India) will be used to measure burden experienced by carers.

Further secondary measures to evaluate the cost effectiveness of the intervention include health-related Quality of Life score via European Quality of Life version 5 (EQ5D-5L) [26] and adapted version of the Client Service Receipt Inventory (CSRI) [27].

The baseline assessment will also include a socio-demographics questionnaire for all participants. Sociodemographics will be summarised as means and proportions.

For all measures, the mean scores at 6 months and 12 months post randomisation will be calculated and compared between the intervention and control group, with the baseline values of each outcome included as covariate.

A subset of 20 patients per country and all 7 clinicians who were randomised to the intervention arm will be invited to attend a qualitative interview after the end of the intervention (6 months) in order to capture the individual experience of the intervention, including barriers and facilitators of attending intervention sessions, suggested adaptations and the practical delivery of the intervention. These interviews will be conducted with the help of a semi-structured topic guide and will be audio recorded. Interviews will be conducted at the end of the intervention (6-month post randomisation) to ensure ease of recollection of the specifics of the intervention, without influencing data collection of the primary outcome.

To maintain blinding of researchers within the study, monitoring of intervention fidelity and the qualitative follow-up interviews will be conducted by researchers who are aware of allocation. The other researchers will conduct the follow-up quantitative assessment and will remain blinded to intervention allocation. Participants and mental health professionals will be asked not to disclose details of the intervention during the follow-up quantitative assessments.

#### Participant timeline {13}

**Fig. 1:**
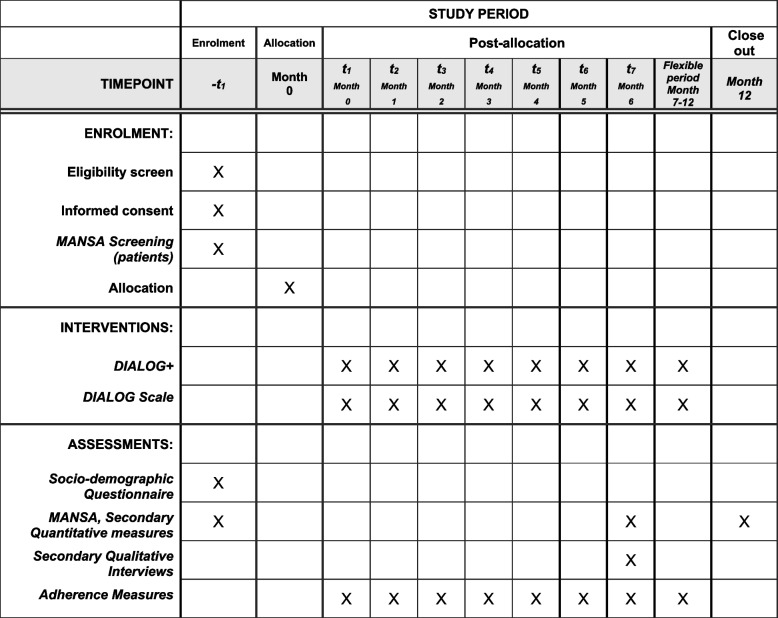
The schedule of enrolment, interventions, and assessments for participants in the DIALOG+ study at all sites in India and Pakistan

A summary of the participant timeline including a schedule for enrollments, intervention sessions and assessments can be found in Fig 1.

### Sample size {14}

#### Quantitative sample size

In order to detect a medium effect size of 0.5, setting power at 90% for 5% significance, accounting for clustering based on an ICC of 0.002 (as observed within the DIALOG+ trial [15]), and applying a conservative design effect of 1.03, the total number of patients required in each country is 84 per group (*n* = 168 per country). After allowing for a drop-out rate of 20%, a total of 210 patients will need to be recruited to give the analysable sample of 168 or 84 per group. Therefore, 14 clinicians will need to be recruited in each country, with an average of 15 patients per clinician.

Clinicians of any background, including lay community workers will be eligible. Meetings held within each of the participating clinical services indicated that there were sufficient numbers of both clinicians and patients, with many outpatient clinics seeing 100+ patients with psychosis per day.

#### Qualitative sample size

Following the 6-month assessment period (primary outcome collection), we will include a purposive sample of 20 participants per country, in individual qualitative interviews to discuss their experience of receiving the intervention. The sampling frame will be comprised of all patients randomised to the intervention arm and will explicitly include participants with positive or negative experiences of the intervention, those whose quality of life improved and those whose did not and those who frequently used or who did not receive the intervention during the testing period. Participant characteristics such as age, gender and clinical site (Pakistan) will also be considered. We will also aim to interview all seven clinicians in each country who delivered DIALOG+ to discuss their experiences of delivering the intervention.

#### Recruitment {15}

In order to ensure adequate participant enrolment, the teams have been actively engaged with the clinical sites. They have held strategy meetings with the management teams at each clinical site before initiation to ensure active involvement from admin and clinical staff. The research teams have set up a stepwise screening and enrolment process. They will actively search through electronic (where available) medical records or individual clinician caseloads to screen for eligible participants with the help of clinical and admin staff at each site. An on-site coordinator will assist with this process. Where patients do not have much time to spare, the research team will split up the consent and screening process to ensure that patients are not missed as a result of time shortage. The research team will also conduct home visits for patients who are unable to complete their assessments at the sites. Recruitment is anticipated to be completed within 6 months however, it is dependent on patient flow at each clinical site.

### Assignment of interventions: allocation

#### Sequence generation {16a}

Permuted blocked randomisation with block sizes of *m* = 4 and 2 will be used within each site. Randomisation will be carried out via an independent statistician via computer generated random numbers to determine allocation. Allocation to intervention and control will be in a ratio of 1:1.

#### Concealment mechanism {16b}

Allocation is only assigned after clusters are complete and all baseline data collected. Randomisation is conducted by independent team members within Queen Mary University of London. Participant allocation is provided only to the unblinded researcher and study coordinator. Allocation status will be saved in password protected electronic folders where they will be inaccessible to blinded researchers.

#### Implementation {16c}

Randomisation will take place after all patients have been enrolled, their baseline data collected and a clinician cluster formed. Randomisation will be conducted by an independent researcher based in the UK. The unblinded researcher will be responsible for informing participants of their allocation. Unblinded researchers/clinical site coordinators will train the clinical staff according to their allocation and schedule future appointments with patients and clinicians. Blinded researchers will not be present at the clinical site while the intervention period/appointment sessions are ongoing to avoid any unblinding.

### Assignment of interventions: blinding

#### Who will be blinded {17a}

Outcome assessors, data analysts and the overall study Principal Investigator will be blinded to the intervention allocation. All documentation revealing allocation will be securely stored and saved so it is inaccessible to blinded researchers. These researchers will have no contact with the participants enrolled and will not visit the clinical facilities when the intervention is taking place.

#### Procedure for unblinding if needed {17b}

In case there is any cause of harm to the participant involved, unblinding is permitted and will be dealt with according to specific safeguarding policies and SOPs in place.

### Data collection and management

#### Plans for assessment and collection of outcomes {18a}

All data is collected by researchers who have been trained in Good Clinical Practice as well as in the individual tools by specialists in their field. Training of clinical measures including BRPS and SANS were done by senior researchers and psychiatrists with previous experience of the measures. For the clinical measures, interrater reliability was established by independently rating videos using the measures and an independent researcher in the UK then calculated inter-rater reliability using the inter-rater correlation coefficient. A high correlation coefficient of over 85% was established and any discrepancies were discussed. Data will be collected on paper case report forms (CRFs). These will then be entered onto an electronic database (REDCap). The REDCap database was developed by the researchers and tested thoroughly at both sites. Data will be cleaned following local standard operating procedures (SOPs). Data collection forms can be available upon request from the authors.

#### Plans to promote participant retention and complete follow-up {18b}

Enrolment logs have been created and will be used to monitor the progress of all participants throughout the trial period until all data has been collected. Within the enrolment log, we monitor all meetings with participants including the date of randomisation, attendance at appointments and completion of each data collection point (baseline, 6 months and 12 months). Any withdrawals or deviation from protocols will be recorded and a note to file/withdrawal form will be added as required. Outcome data will be collected from all participants unless they have explicitly asked us not to contact them further. For participants who discontinue or deviate from intervention protocols, a purposive sample of qualitative interviews will be conducted at 6 months to understand any reasons for this. Sampling will be based on clinical site, gender and time of discontinuation/deviation.

Participants will receive reminder calls before each appointment and will be contacted in advance of any research assessments. Researchers schedule home visits to complete follow-up assessments where necessary. Participants will receive compensation for transport and the time taken for the data collection appointments (PKR 500 for screening, PKR 750 for complete assessment for patients and PKR 500 for caregivers in Pakistan and INR 300 for screening, INR 400 for complete assessments and INR 500 for caregivers in India).

#### Data management {19}

All data will be collected on paper based CRFs and safely stored within locked cabinets at the main trial site. Data from the CRFs will be quality checked by the local trial project manager (PM), as soon as possible. Any errors will be resolved with the individual who initially completed the CRF and verified against source documents where possible. All data will then be entered on electronic data collection tools designed on REDCap. The database has inbuilt validations to ensure data entry is as accurate as possible and during database development it went through multiple iterations of testing and feedback before finalisation to ensure smooth data entry operations. Data entered on REDCap will be periodically checked by the PM and against the source CRF to ensure there are no discrepancies. The team from the UK will also conduct regular monitoring visits and randomly check 10% of all the data collected and entered to ensure data quality is maintained. Data management procedures can be found in the ‘Document completion, Transport and Storage SOP’ and ‘Data Entry SOP’ created by each site. This is available upon request.

### Confidentiality {27}

#### Personal information

All data will be pseudonymised to maintain patient confidentiality. All participants will be assigned a participant ID number used for all data processing purposes. Patient identifiable data (participants’ names, contact details, sociodemographic data) and the list linking these data with the participant ID number will be stored on computers using a secure drive, within password-protected folders, which will only be accessible to the research team. All hard copies of data including socio-demographic forms, consent forms and patient receipts will be kept in lockable filing cabinets within premises of SCARF (India), JPMC (Pakistan), and KeH (Pakistan) or Interactive Research and Development (Pakistan) and only accessible by the research team.

To further protect confidentiality, we will:

1. Ensure that participants understand during the informed consent process where interviews and focus groups might be audio-recorded, the purpose for the audio-recording, how the audio files will be stored, and who will have access to these files.
2. Remind all participants that they do not have to answer any questions or make any personal disclosures if they do not wish to.
3. Refrain from using participants’ names and the names of mental health services and during audio-recorded interviews. Participants will be reminded to refrain from using personal names or locations where possible at the start of the interview.
4. Consent forms containing personal details of participants will be kept completely separate from the CRFs.

Where the researcher has concerns regarding the participant’s safety or the safety of others, through participant disclosures of thoughts/plans of harming themselves or others, then the researcher is obliged to break confidentiality and inform the relevant clinical teams, services and/or authorities. This will be made clear to the participant on the information sheet during the consent process to ensure their understanding.

All investigators and study staff will comply with the requirements of the Law 1581 (2012) and Decree 1377 (2013) and any other associated legislation of India and Pakistan regarding the collection, storage, processing, and disclosure of personal information and will uphold the law’s core principles throughout the study.

#### Audio recordings

The focus groups and individual interviews will be audio-recorded using an encrypted device with consent from participants. Audio recordings will be stored in password-protected folders on computers using a secure drive, which will only be accessible to the research team. The audio recordings will be destroyed immediately after transcription. All transcriptions will be completed by a professional transcription service. Prior to transcription, all identifiable information will be removed and/or replaced with pseudonymised labels.

#### Record retention and archiving

Research data will be retained and archived in accordance with the Research Governance Framework and IM&T Information and security policies. Records will be archived as per Queen Mary University of London procedures and kept for 20 years in the Trust Modern Records Centre. The PI (Bird) will be the custodian of the data.

The data will also be stored at the main study site in India (SCARF) where Ramachandran will be custodian of the data and main study sites in Pakistan (JPMC and KeH) where Pasha will be custodian of the data. This will be done according to the local regulations for data storage and protection.

Electronic data will be entered on a secure REDCap server in India and Pakistan and will be shared with the research team in the UK.

#### Plans for collection, laboratory evaluation and storage of biological specimens for genetic or molecular analysis in this trial/future use {33}

Not applicable as no samples collected.

### Statistical methods

#### Statistical methods for primary and secondary outcomes {20a}

A statistical analysis plan will be discussed and agreed with the local research team and the International Steering Committee (ISC) prior to data analysis. The local research team will take a leading role in the management and analysis of data. Data analysis will not be conducted prior to sign off and blinded members of the research team, including the statistician responsible for the analysis, will remain blinded until sign off has been obtained.

#### Quantitative data analysis

The number of screened participants, eligible participants, and of those who refused participation or were not approached will be recorded. The analysis will assess the number of intervention sessions received by participants, and we will collect data on drop-out (including reasons for drop-out if available) from treatment. The intervention topics discussed in meetings will be tabulated.

Descriptive statistics will be reported for socio-demographic data for all participants. Nominal and ordinal data will be presented as proportions (frequency), whereas continuous variables will be described utilising central tendency and variability measures. The latter will be selected according to distributions found (parametric or non-parametric), which will be evaluated through normality tests and histograms (probability functions). To assess the effectiveness of DIALOG+, mean and standard deviations (or median and interquartile range) over the three time points (baseline, 6 months and 12 months) will be calculated, and analysis will test the significance of the differences between the means (or other parameters) of outcomes measured. The primary outcome analysis will be the comparison of mean MANSA scores between treatment groups at 6 months follow-up using a mixed-effects model. This model will account for clustering by clinician, baseline values of the outcome (MANSA) and site (Pakistan) as covariates. A full analysis plan will be developed prior to data analysis, which will consider which covariates should be adjusted for in the model and methods for dealing with missing data.

The analysis will be conducted on an intention-to-treat basis, and every effort will be made to collect complete data. Patterns of missing data will be explored, and a strategy for dealing with missing values will be developed in the formal statistical analysis plan. Methods for dealing with missing data will depend on the extent, type and distribution of missingness.

#### Economic evaluation

The objective of the economic evaluation will be to inform decision-makers in India and Pakistan about the incremental cost and associated health outcomes of using DIALOG+ in the routine management of psychosis relative to the active control and from the perspective of the healthcare system. The supplementary objective of the economic evaluation will be to generate evidence on the costs of including DIALOG+ in the existing healthcare delivery systems in public and not-for-profit private sectors in India and in Pakistan.

The economic component will collect data on costs and health outcomes associated with the intervention and control arms. Costs will cover health sector costs and resources associated with providing the intervention as well as cost to the patient and their families for seeking care. Methods of micro costing will be used to determine the monetary value of the resources used for the management of psychosis with DIALOG+ and the active control. Healthcare cost will include time cost of healthcare providers, cost of capital resources and their maintenance and cost of supplies. A time and motion study will be conducted to estimate the resources use of the health facilities [28]. Purchase prices of all resources including supplies, instrument and furniture will be obtained from the respective health facilities where applicable, alternatively a market survey will be carried out. Data on resource use of the participants will be obtained from the customised CSRI to cover a retrospective period of 6 months prior to baseline and the two 6-month follow-up periods.

Health outcomes will be measured in quality-adjusted life years (QALYs). The European quality of life instrument EQ5D-5L will be used to capture health effects on patients, alongside the MANSA. Urdu and Tamil translation of EQ5D-5L will be obtained from the EUROQOL group. The EQ5D-5L tool will be administered at baseline, 6 months and 12 months follow-up. The valuation set of preference of the general population required to convert the EQ5D-5L to QALYs will be obtained from studies conducted in India [29] whereas the value set of EQ5D-5L for Pakistan is completed and ready for publication. The difference in outcomes, i.e. QALYs at the end and baseline of RCT, will demonstrate effectiveness for each arm of study and will result in the calculation of incremental health benefits of DIALOG+.

Incremental cost effectiveness ratios (ICERs) will be calculated as the ratio of difference in costs over difference in outcomes.

Uncertainty will arise in the estimates of cost, QALYs and ICERs from two sources (1) sampling and (2) methodological assumptions. Methods similar to those for the RCT will be used in the economic evaluation to deal with missing data or censored data. Skewness and correlation in cost and outcomes data will be treated with Bayesian or frequentist techniques. For Pakistani results, ICERS will be adjusted for clustering effect at facility level. Probabilistic one-way or two-way sensitivity analysis will be carried out as a robustness check for the uncertainty surrounding the estimated ICERs due to assumptions used while estimating costs and outcomes.

A health economic analysis plan will be developed and agreed with the local research team and signed off by the International Steering Committee (ISC) prior to data analysis.

#### Qualitative data analysis

For the process evaluation interviews and focus groups, thematic analysis following the guidance of Miles & Huberman [30] will be conducted using NVivo qualitative analysis software. All interviews will be audio-recorded and transcribed verbatim. A researcher will remove all identifying information from the transcripts, including any references to patients, clinicians or local services.

An inductive approach will be used to provide new insights and a richer understanding of the data without using preconceived categories. Two members of the research team will first familiarise themselves with the transcripts. Open coding will be used (making notes and headings in the text to describe the content). Similar codes will be grouped under themes, and the identified themes and sub-themes will then be checked and refined. Inter-rater reliability in applying second level codes (or categories) will be calculated for 20% of the data.

#### Interim analyses {21b}

No interim data analysis is currently planned.

#### Methods for additional analyses (e.g. subgroup analyses) {20b}

Planned sensitivity and subgroup analyses will be outlined in the statistical analysis plan and signed off by the statistician and ISC prior to analysis.

#### Methods in analysis to handle protocol non-adherence and any statistical methods to handle missing data {20c}

Where data are missing for the primary outcome (MANSA at 6 months), individual level single imputations in which missing values are replaced by a fixed value following a predetermined rule will be used. The conditions in which imputation will occur will be specified in the statistical analysis plan, including circumstances where data would not be imputed (for example, the patient is deceased). Sensitivity analyses will be conducted to assess the impact of missing data under reasonable assumptions about the behaviour of those lost to follow-up. In each case, analysis will be carried out on the subset of participants for whom the outcome in question has been recorded and model estimates compared against analogous estimates from the imputation analysis to ascertain the impact of loss to follow-up in this study.

#### Plans to give access to the full protocol, participant level-data and statistical code {31c}

Following publication of the main paper, the full anonymised participant-level dataset will be made available on request, subject to written approval from the ISC.

### Oversight and monitoring

#### Composition of the coordinating Centre and trial steering committee {5d}

The PIECEs trial’s main coordinating centre is QMUL in the UK, where the PI, an overall programme manager and a trial manager provide technical, financial and programmatic support to the partner sites and provide day to day guidance, support and training to the teams to execute the trial at both sites.

The teams are further supported by the ISC which meets biannually to discuss project achievements, challenges and next steps. The ISC is made up of members from each partner country and the UK who provide oversight and expertise in the areas of clinical psychiatry, health systems, community engagement and outreach, and lived experience of psychosis.

#### Composition of the data monitoring committee, its role and reporting structure {21a}

The ISC will also take on the role of the DMC with the addition of an independent statistician as the team work further to develop the statistical analysis plan and begin data analysis.

#### Adverse event reporting and harms {22}

##### Adverse events (AE)

Any adverse events will be recorded in the main research file and the participant’s clinical records, if appropriate, and the participant followed up by the research team.

##### Serious adverse event (SAE)

SAEs that are ‘related’ and ‘unexpected’ will be reported according regulations of the Research Ethics Committee (REC) and other relevant regulations in India and Pakistan.

##### Urgent safety measures

In the case of urgent safety measures being required, the local PI will inform the UK PI and the REC of the event as per REC and other relevant requirements and guidelines.

##### Annual safety reporting

The local PIs will send over annual reports as required by the REC using their existing templates and guidelines.

##### Overview of the safety reporting responsibilities

The local PIs will ensure that safety monitoring and reporting is conducted in accordance with the requirements of the REC and any other relevant organisations/institutions that are involved in overseeing and monitoring research activities in India and Pakistan.

#### Frequency and plans for auditing trial conduct {23}

Members of the UK-based research team will carry out monitoring visits to ensure that research activities are being implemented as outlined in agreed protocols and SOPs and in accordance with ethical guidelines and data protection legislation. This will include checking the secure storage of electronic and paper records/data, the correct completion of agreed recruitment and enrolment logs and case report forms and accurate data entry.

Further monitoring, audit and inspection will be carried out annually by independent local research bodies within the local institutions.

#### Plans for communicating important protocol amendments to relevant parties (e.g. trial participants, ethical committees) {25}

Any amendments which take place after the trial has begun will be submitted to SCARF (India), the HMSC of the ICMR (India), Jinnah Postgraduate Medical Centre (Pakistan), Karwan-e-Hayat (Pakistan), IRD IRB (Pakistan) and QMUL REC for approval and the amendment history will be tracked via version and date control of protocols.

## Dissemination plans {31a}

The aims and impact of the RCT will be achieved through a comprehensive communication plan, which will inform the different stakeholders of the research findings and programme outputs. This will ensure that the new knowledge obtained translates into improved health outcomes. Target audiences include (but are not restricted to) people with psychosis and their family members, service commissioners, hospital managers, policy-makers, clinicians, academics and the public. Dissemination activities will include the following:

1. A project specific website has been developed and is regularly updated and we utilise social media (including Twitter, Instagram and blogs) to communicate research findings to a wider audience. Social media within India and Pakistan is increasingly popular as a way of mass communication. We will ensure information is available in different written and visual formats and will include videos outlining the main findings to be uploaded to portals such as YouTube and Instagram to increase reach.
2. The adapted DIALOG+ intervention will be available as an app in the local languages as well as in English. Both the app and training package will be freely available online via the project specific website and the partner organisation websites. The app will be available to download via the app store and play store. Dissemination and knowledge exchange activities focused on services, policy-makers and researchers will increase access to and awareness of these materials.
3. Findings will be published in high-impact peer-reviewed open access journals to maximise impact. Clinicians and early career researchers from Pakistan and India will be encouraged to take the role of lead author as part of the capacity building process.
4. Newsletters and user publications will be produced for clinicians, patients and the public, which will aim to be engaging and appealing to both professionals and lay people. The Lived Experience Advisory Panel will be actively involved in developing lay summaries and ensuring the content is understandable to the local audience.
5. To launch the findings of the intervention and all associated materials, we will hold dissemination and knowledge exchange events inviting different stakeholders including academics, clinicians, policy-makers, service managers, patients, carers and the local community.
6. Senior and junior researchers will be encouraged to present findings at national and international conferences.
7. The co-applicants and ISC will use their existing networks (with links in commissioning, policy-making, quality improvement and service development) to disseminate the findings and online materials. The two WHO collaborating centres (SCARF and QMUL) will make use of this status and actively engage the regional officers in the dissemination activities.

## Discussion

This study addresses the treatment gap for individuals with SMI, in India and Pakistan, by utilising the existing resources within the healthcare system as well as mobilising the resources of patients, their families and communities. It aims to test a low-cost, resource-orientated approach to improve the quality of community-based care for people with psychosis in two cities in India and Pakistan. The intervention to be tested is evidence based and is generic, such that it can be used by a range of staff in different clinical settings without the need to set up new services or employ specifically trained staff. The inclusion criteria for the trial is broad to reflect the individuals commonly seen in community-based services. This will allow us to assess a range of patient-level (e.g. financial and clinical status) characteristics which might influence intervention effectiveness. The active control condition will help control for the additional time during the consultation and for the addition of a tablet computer, which may be novel within these LMIC settings.

Despite the strengths of the study, there are a number of limitations to consider.

Karachi and Chennai are both cosmopolitan urban sites within Pakistan and India where there are a variety of local languages spoken by the residents. Being larger cities with greater resources, many patients will travel to the cities from neighbouring villages to seek mental health services and medical help. Our study excludes patients who do not speak the most common local language which reduces the population we can recruit, however, if the intervention proves effective it can easily be further translated for future use more broadly.

To ensure the trial runs as per the protocol, researchers play a big role in facilitating the process of appointments, including providing frequent reminders and rescheduling of missed appointments to ensure we are able to test the intervention as it is proposed for the first 6 months of the trial. We overcome this limitation to a great extent by observing if/how patients and clinicians manage their own appointments and sessions for the period between the 6- and 12-month follow-up to understand how the intervention would work without added assistance.

India and Pakistan have a large proportion of the population which resides within rural settings, which limits the generalisability of our findings. However, if the intervention is found to be effective in improving the quality of life for individuals with long-term psychosis in these urban settings, there is potential for the approach to be adopted within rural settings and other LMICs, where a treatment gap exists for individuals with SMI. The flexibility of the approach, such that it can be delivered by a range of professionals, with only minimal training, will facilitate uptake and wider implementation across different clinical settings, ultimately improving the quality of life for millions of individuals with long-term psychosis worldwide.

## Trial status

Protocol version: V1.0, 15 January 2022

Date recruitment began: in India—21 April 2022, in Pakistan—7 June 2022

Date recruitment is expected to be completed: 31 December 2022

## Data Availability

The final trial dataset for each partner country will be held within the main partner sites (IRD in Pakistan and SCARF in India) and the full trial dataset including data from both sites will be held by the PI in the UK at QMUL (Bird).

## Abbreviations

SMI: Severe mental illness
QoL: Quality of life
RCT: Randomised controlled trial
LMICs: Low- and middle-income countries
HICs: High-income countries
SCARF: Schizophrenia Research Foundation
JPMC: Jinnah Postgraduate Medical Centre
KeH: Karwan-e-Hayat
MANSA: Manchester Short Assessment of Quality of Life
UBACC: University of California, San Diego Brief Assessment of Capacity to Consent
BPRS: Brief Psychiatric Rating Scale
SANS: Scale for Assessment of Negative Symptoms
WHODAS: World Health Organization Disability Assessment Schedule
SIX: Social Situation Index
HAS: Helping Alliance Survey
EQ5D-5L: European Quality of Life version 5
CSRI: Client Service Receipt Inventory
CRFs: Case report forms
SOPs: Standard operating procedures
PM: Project manager
IRD: Interactive Research and Development
ISC: International Steering Committee
QALYs: Quality-adjusted life years
ICERs: Incremental cost effectiveness ratios
WTP: Willingness to pay
QMUL: Queen Mary University of London
